# Construction Notes

**Published:** 1896-07-11

**Authors:** 


### CONSTRUCTION NOTES.

### Lightburn Joint Fever Hospital.

This hospital, which has been erected near Shettleston, for the accommodation of patients from the lower and middle ward districts of Lanarkshire, has now been formally opened. The building has been erected at the joint expense of the district committees of these wards, at a total cost estimated at £29,000. Here ample accommodation is provided for sixty beds. Messrs. James M. Thomson, and Messrs. R. Thornton, Steele, and Thomson, architects, of Edinburgh, prepared the plans for the buildings. The site forms an oblong, lying N. and S., with entrance gate, porter's lodge, and waiting room on the south side of Cavatyne Road. To the left on entering are the offices, where are situated the engines and boilers, the laundry, the stables, workshops, the steam disinfecting rooms, and the refuse destructors. In the centre of the grounds, facing the entrance gateway, are the administrative buildings, consisting of two storeys with attics. There is a kitchen in the rear; in these buildings is accommodation for the hospital physician, the matron, fourteen nurses, and ten servants. On either side of the block is an observation ward, in each of which there are two beds. In the southern half of the grounds are four pavilions, completely isolated from one another. Two of these have each seventeen beds, one pavilion being for cases of typhoid fever and the other for diphtheria. There are two pavilions, each containing eleven beds, for cases of scarlet fever. In the common wards each patient has 145 square feet of floorage and 2,000 cubic feet of air space. An underground tunnel connects the various pavilions with the offices, and in it are laid the hot water, drainage, and other pipes, as well as the electric cables. The ceilings of the pavilions are arched up into the roof, and provided with dormer windows. In addition to the usual lavatory accommodation, each pavilion has a suite of bath and dressing rooms. Heating is effected by a system of low-pressure hot-water pipes, in addition to an open fire in each ward. Electric light is used throughout the building.

